# Conformational Changes of Calmodulin on Calcium and Peptide Binding Monitored by Film Bulk Acoustic Resonators

**DOI:** 10.3390/bios1040164

**Published:** 2011-12-14

**Authors:** Martin Nirschl, Johannes Ottl, Janos Vörös

**Affiliations:** 1 Laboratory of Biosensors and Bioelectronics, Institute for Biomedical Engineering, ETH Zurich Gloriastrasse 35, 8092 Zurich, Switzerland; E-Mail: janos.voros@biomed.ee.ethz.ch; 2 Novartis Institute of Biomedical Research Basel, CPC/LFP, Novartis Pharma AG, Postfach, Basel CH 4002, Switzerland; E-Mail: johannes.ottl@novartis.com

**Keywords:** FBAR, film bulk acoustic resonators, calmodulin, calcium, conformational change

## Abstract

Film bulk acoustic resonators (FBAR) are mass sensitive, label-free biosensors that allow monitoring of the interaction between biomolecules. In this paper we use the FBAR to measure the binding of calcium and the CaMKII peptide to calmodulin. Because the mass of the calcium is too small to be detected, the conformational change caused by the binding process is measured by monitoring the resonant frequency and the motional resistance of the FBAR. The resonant frequency is a measure for the amount of mass coupled to the sensor while the motional resistance is influenced by the viscoelastic properties of the adsorbent. The measured frequency shift during the calcium adsorptions was found to be strongly dependent on the surface concentration of the immobilized calmodulin, which indicates that the measured signal is significantly influenced by the amount of water inside the calmodulin layer. By plotting the measured motional resistance against the frequency shift, a mass adsorption can be distinguished from processes involving measurable conformational changes. With this method three serial processes were identified during the peptide binding. The results show that the FBAR is a promising technology for the label-free measurement of conformational changes.

## 1. Introduction

Many proteins undergo conformational changes upon the binding of a ligand. This change is mostly a change in the tertiary structure of the protein or involves rearrangements of parts of it, e.g., helices, turns or pockets. Because typically only small parts of the proteins (the domains) are involved in a certain function even a small conformational change can cause significant changes in the protein’s functional activity. Controlling this activity e.g., of protein kinases is of interest in for example cancer therapy [1]. 

A molecule, which is known to undergo conformational changes, is calmodulin [2]. A wide range of technologies was employed to investigate the conformational changes of calmodulin. There are solution based technologies like mass spectrometry [3], NMR [4], gel electrophoresis [5], fluorescence resonance energy transfer (FRET) [6], small angle X-ray scattering [7], circular dichroism [8], calorimetry [9] or backscattering interferometry (BSI) [10]. More recently, surface based technologies like dual polarisation interferometry (DPI) [11], optical second-harmonic generation (SHG) [12], surface acoustic wave devices [13,14] or the quartz crystal microbalance (QCM) [15,16] were employed. 

Each of them has specific advantages and disadvantages: Solution based transducers generally require a larger sample amount but the information obtained has a high quality, e.g., NMR can be used to measure the position of the atoms in the molecule with a very high precision if isotope labeled protein is available and hence residue assignment can be achieved. Some technologies allow only static measurements and if at all only with high effort in real-time (X-Ray crystallography and NMR). For FRET it is necessary to add two suitable labels to the protein. QCM allows label-free measurements in real-time but the large sensor size imposes difficulties in integrating a high number of sensors in one multiplexed device. 

In this paper we evaluate the possibility of investigating conformational changes using film bulk acoustic resonators (FBAR). While FBARs have been used as frequency filters for decades [17], their usage as a biosensor is rather novel [18]. We previously reported the usage of the FBAR for the label-free detection of proteins [19] and DNA [20]. We also showed that the FBAR is highly sensitive to viscoelastic properties of the adsorbent [21]. 

Here we use the FBAR to investigate the response of the resonant frequency and the motional resistance to the conformational changes of calmodulin on calcium and peptide binding. We show that the FBAR is highly sensitive to the conformational changes of calmodulin and that it is possible to distinguish between mass adsorption and conformational changes. 

## 2. Experimental Section

### 2.1. FBAR and Read-Out

The FBARs used in this study consist of a 500 nm thick piezoelectric ZnO layer sandwiched between two electrodes. The resonators were mounted on top of an acoustic mirror in order to avoid acoustic energy loss into the substrate. A 300 nm thick SiO_2_ layer electrically isolated the resonator from the surrounding liquid. A 25 nm thick gold layer sputtered on top of the SiO_2_ serves as an interface to the surface chemistry. The lateral size of one sensor surface was 200 µm × 200 µm. The gold surface on the FBARs was cleaned in oxygen plasma for 5 min at 100 W before the measurements. The impedance of the resonators was continuously measured over the electrodes in a frequency range near the resonant frequency at 800 MHz using a network analyser (Agilent 8720ES). The serial resonant frequency was determined by searching the frequency with the highest conductance. The admittance at resonant frequency was used as a measure for viscoelastic dissipation. We assume that the change in admittance represents mainly a change in the motional resistance, which is a measure of the energy dissipated by the adsorbent [16]. As a consequence the measured change in admittance at serial resonant frequency is named motional resistance throughout this paper. 

A flow cell (about 60 µL volume) was mounted on top of the FBAR; all measurements were carried out at a flow of 10 µL/min using a peristaltic pump. When changing liquids the flow was increased to 900 µL/min for 10 s to ensure a compete exchange of the liquids in the flow cell. 

A mass sensitivity of 4 kHz cm^2^/ng was used to convert from frequency shift (e.g., kHz) to surface mass coverage (e.g., ng/cm^2^) if not stated differently. The production of the ZnO, the description of the FBAR, the fluidic cell and the read-out were described in detail elsewhere [22,23]. 

### 2.2. Reagents and Materials

Neutravidin was obtained from Pierce (Rockford, IL, USA). Biotinlyated calmodulin and Ca^2+^/calmodulin kinase II inhibitor (CaMKII 281-309) were purchased from Calbiochem (San Diego, CA, USA) and used within two weeks of thawing. Biotin, HEPES, NaCl, CaCl_2_ and EDTA were all purchased from Sigma Aldrich (Germany). In all measurements 10 mM HEPES-NaOH, pH 7.2, 100 mM NaCl was used as buffer. 

## 3. Results and Discussion

### 3.1. Immobilisation of Neutravidin and Biotinylated Calmodulin

**Figure 1:**
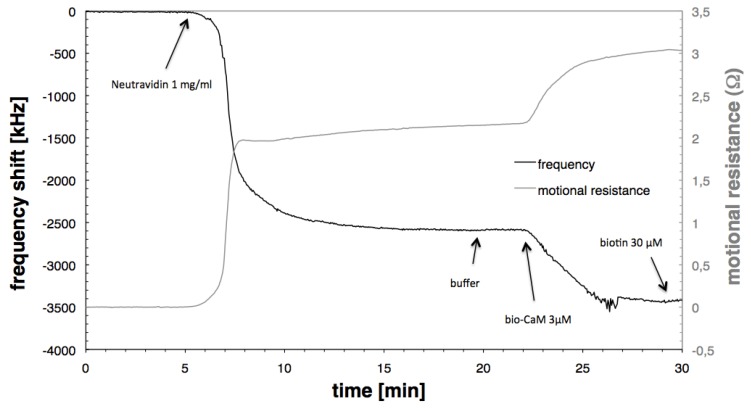
Resonant frequency shift and motional resistance change during Neutravidin adsorption and binding of biotinylated calmodulin.

In order to measure interactions between calmodulin and other molecules the calmodulin has to be immobilised on the sensor surface in such a way that the calmodulin is able to chemically react similarly to the way it would react in solution. From literature it is known that this is the case if calmodulin is bound to immobilised Neutravidin using biotin as a linker [15,16]. Neutravidin (1 mg/mL) was adsorbed in flow directly onto the cleaned gold surface. The binding of the Neutravidin to gold could be either attributed to physisorption or to sulfur-metal interaction [16,24]. After a buffer rinse, biotinylated calmodulin in concentrations of 30 nM, 300 nM and 3 µM was added. Figure 1 shows the typical frequency and motional resistance behaviour during the Neutravidin adsorption and subsequent biotinylated calmodulin binding. For both processes a decrease in frequency indicating the adsorption of mass to the surface occurs concurrently with an increase in motional resistance, which reflects an increase in dissipation caused by the viscoelastic character of the adsorbed molecules. Biotin (20 µM) was then added to saturate the remaining neutravidin binding sites.

Table 1 summarises the adsorbed bio-CaM at three concentrations. At the highest concentration (3 µM) a surface mass of 190 ng/cm^2^ bio-CaM (*i.e.*, 11.4 pm/cm^2^) was immobilised on the Neutravidin surface. This corresponds to 1.17 molecules of calmodulin per immobilised Neutravidin. 

**Table 1:** Summary of results for the bio-CaM binding and CaCl_2_ adsorption of the FBAR measurements together with values from QCM literature ([15,16]).

Measured on	Concentration bio-CaM [nM]	Bio-CaM bound to surface [ng/cm^2^]	Binding efficiency of bio-CaM to Neutravidin	Mass change on addition of 1 mM CaCl_2_ [ng/cm^2^]	Ratio surface mass CaCl_2_/bio-CaM
FBAR	30	79	0.45	10	12.7%
FBAR	300	100	0.69	6	6.0%
FBAR	3,000	190	1.17	−36	−19.1%
QCM (Data from [15])	30	111	0.7	6	5.5%
QCM (Data from [16])	3,000	42	n.a.	10	23.1%

### 3.2. Calcium Induced Conformational Changes of Calmodulin

**Figure 2:**
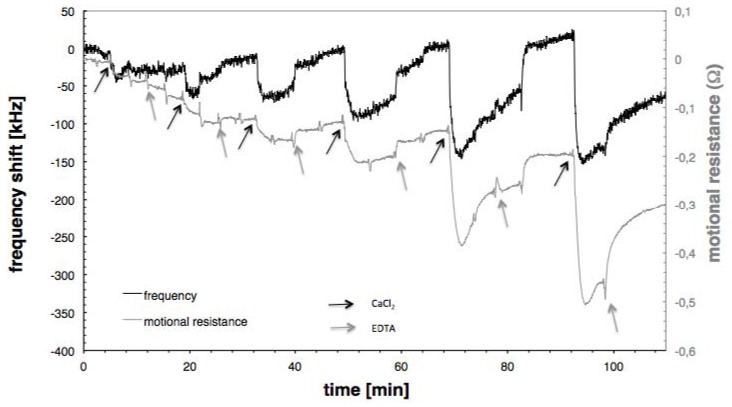
Frequency and motional resistance caused by the calcium adsorption in increasing concentrations. Black arrows show the injections of CaCl_2_, grey arrows the injections of EDTA injections.

CaCl_2_ was injected in increasing concentrations onto the calmodulin surface while monitoring the resonant frequency and the motional resistance. EDTA was used to de-bind the calcium from the calmodulin. Figure 2 shows the frequency and motional resistance change during calcium adsorption and desorption for the highest calmodulin surface concentration (190 ng/cm^2^). Upon calcium adsorption (black arrows) both the frequency shift (black line) and the motional resistance (grey line) decrease. With the addition of EDTA the resonant frequency is restored to the original level and the motional resistance goes back close to the original level with a slight drift downwards. The behaviour of the motional resistance was similar (*i.e.*, decrease with calcium adsorption) for all calmodulin concentrations. This decrease of the motional resistance is due to a decrease in acoustic energy dissipated in the calmodulin layer. From the literature it is known that calmodulin transfers from a flexible to a more rigid molecule when calcium is bound and thus adsorbs less acoustic energy [25]. 

In two reference measurements, calcium and CaMKII peptide were injected onto a biotin saturated Neutravidin surface; the change in frequency and motional resistance was below the noise level in both cases. 

While the behaviour of the motional resistance was qualitatively equal for all calmodulin surface concentrations, the behaviour of the resonant frequency was found to be highly dependent on the surface concentration of calmodulin. In order to investigate the concentration dependence, the ratio of the frequency shift observed during calmodulin adsorption and the frequency shift observed during calcium adsorption was calculated for all measurements. This measure shows the frequency shift per amount of calmodulin and thus would be constant if the signal at calcium adsorption was linearly dependent on the amount of available calmodulin on the surface. 

**Figure 3:**
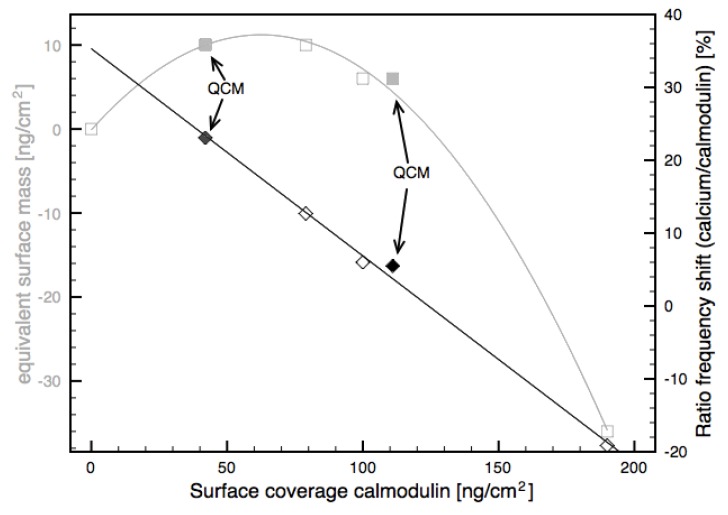
Frequency response caused by calcium adsorption for different surface densities of calmodulin. Ratio of the frequency shift of the calcium adsorption to the frequency shift of the calmodulin adsorption is plotted in black. Equivalent surface mass calculated from the frequency shift is plotted in grey. Values for the QCM were taken from [15,16]. A line was fitted through the points showing the ratio of the frequency shifts and parabola through the points showing the equivalent surface mass.

In Figure 3 the ratio of the frequency shift caused by the adsorption of calcium at 1 mM to the frequency shift of the calmodulin adsorption is plotted against the surface density of the calmodulin (black lines and symbols). The equivalent surface mass, obtained by converting the resonant frequency shift of the calcium adsorption into mass per area assuming that the frequency shift is only influenced by mass changes on the surface is also shown (grey lines and symbols). For calmodulin densities lower than around 120 ng/cm^2^, the frequency shift is positive on both QCM and FBAR. Because the shift is positive this cannot be explained with the mass of the bound calcium because an increase of mass causes a negative frequency shift on QCM and FBAR. The positive shift is likely to be the result of a conformational change as suggested previously [15,16]. We suggest that a change in the tertiary structure in the calmodulin decreases the amount of water bound to the calmodulin and causes the increase in the resonant frequency. 

For the highest surface density (190 ng/cm^2^) the frequency shift is negative, indicating that more mass is bound to the sensor surface. This mass bound to the surface, however, cannot be only the mass of the adsorbed calcium, which is too small to significantly contribute to the shift. The mass effect of calcium bound to all 4 binding sites of all available calmodulin molecules would contribute only −8 kHz to the frequency shift, whilst the measured change is −168 kHz. We therefore suggest that both the negative and the positive frequency shift are caused mainly by changes in the mass of water that moves together with the calmodulin. Coupled water within biomolecular layers is known to significantly contribute to the frequency shift on acoustic resonators [26,27,28,29]. The coupled water also explains the behaviour of the frequency shift: At a lower protein surface density, the amount of water coupled inside the biomolecular layer is higher than at high surface concentrations. As an example, the ratio of the mass of the bound water to the mass of adsorbed streptavidin was found to vary from 7 to 1.5 with increasing surface density [30]. Similar conditions might be true for the calmodulin layer. Unfolding of the calmodulin molecule which exposes hydrophobic domains on calcium binding [31,32] is likely to decrease the water content inside the layer, causing an increase of the resonant frequency. For higher calmodulin surface concentrations this effect decreases due to the lower water content of the calmodulin layer. On the contrary, the prolongation of the calmodulin length upon calcium binding [9] increases the amount of water in the surface through certain effects: firstly, the increasing thickness of the layer causes a larger water layer to be moved together with the acoustic resonator and secondly, the mass density of the layer is decreased by the elongation and in this way more space is made available for water molecules. In addition, the pure increase of the thickness of the calmodulin layer alone will increases the wavelength of the acoustic wave in the resonator. The frequency shift of the elongation of a protein layer from 5.8 nm to 6.2 nm was calculated to be −36 kHz using the model by Mason [33,34]. This implies that together with the −8 kHz shift from the calcium mass −124 kHz would result from a change in coupled water. 

At a calmodulin surface concentration of around 120 ng/cm^2^ the amount of coupled water within the layer remains constant *i.e*, the increase of coupled water due to an increase in layer thickness and the decrease due to the exposure of hydrophobic domains fully compensate, resulting in a zero resonance frequency shift. 

**Figure 4:**
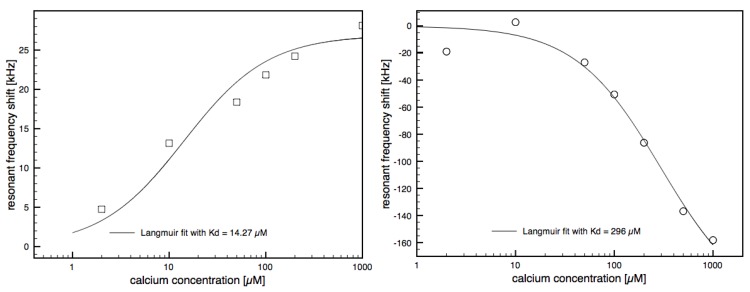
Frequency shift of calcium binding to calmodulin at different calcium concentrations for the two different surface concentrations of 100 ng/cm^2^ (**left**) and 190 ng/cm^2^ (**right**). The line shows the results of the Langmuir isothermal plots with fitted *K_d_* of 14.27 µM and 296 µM.

At calmodulin surface concentrations of 100 and 190 ng/cm^2^, the titration curves for calcium concentrations ranging from 2 µM to 1 mM were measured. Figure 4 shows the titration curve for a calmodulin surface concentration of 100 ng/cm^2^. The line shows the results of a fit of the data to a Langmuir isotherm fit, to obtain the apparent dissociation constant *K_d_*. The *K_d_* obtained for the surface concentration of 100 ng/cm^2^ was 14.27 µM, within the range from 3 to 22 µM for the four different calcium binding sites reported in the literature [35]. The *K_d_* using the highest surface concentration of the calmodulin was measured to be 296 µM and thus one order of magnitude higher than the values from the literature. Apparently, the *K_d_* is overestimated if the surface concentration is too high. One reason for this might be steric hindrance. For accurate *K_d_* determinations the surface density of the calmodulin should be therefore not too high but still in a range where the obtained frequency shifts are significant enough to be measured. Judging from Figure 3, calmodulin surface density of around 70 ng/cm^2^ would be expected to give reasonable *K_d_* results together with a reasonable signal-to-noise ratio of the resonant frequency. 

### 3.3. CaMKII Binding

**Figure 5:**
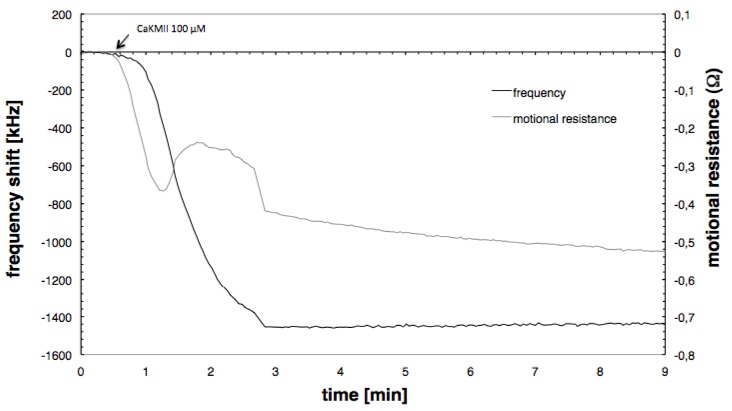
Frequency response and motional resistance change of CaMKII peptide binding to the Ca^2+^/calmodulin complex.

Figure 5 shows the binding of the peptide CaMKII to ApoCaM at a calmodulin surface concentration of 190 ng/cm^2^. The binding curve was qualitatively the same at all concentrations but the signals were the highest at this concentration. The arrow indicates the point at which the peptide was injected. Both the buffer and the CaMKII solution contained 1 mM CaCl_2_. 

The injection of the peptide is followed by a decrease of both frequency and motional resistance. However, the change of motional resistance starts promptly after the injection, whereas the frequency changes follows with a delay of about 45 s. This behaviour indicates that the mass adsorption is preceded by a conformational change of the calmodulin. While the resonant frequency shows a normal binding behaviour, as seen for Neutravidin or calmodulin adsorption, the initial decrease of the motional resistance stops and reverses for about 90 s, before again beginning a downward progression. A further conformational change causing changes in the viscoelastic properties of the adsorbed molecules might be the origin of this behaviour. The resonant frequency shift at saturation (−1.4 MHz) is too large to be caused purely by the mass of the peptide. The frequency shift expected when one peptide binds to each calmodulin molecule on the surface is only −161 kHz. The additional contribution might be either caused by the peptide increasing the amount of water within the calmodulin layer or by a large amount of water coupled to the peptide itself. 

### 3.4. Conductance *versus* Frequency Shift Plots

In an attempt to show the adsorption processes in an easier and more comprehensible way, figures plotting motional resistance against frequency shift are used to describe the Neutravidin adsorption and subsequent biotinylated calmodulin binding, the binding of calcium to and removal from the calmodulin and the binding of the peptide to the Ca^2+^/calmodulin. 

**Figure 6:**
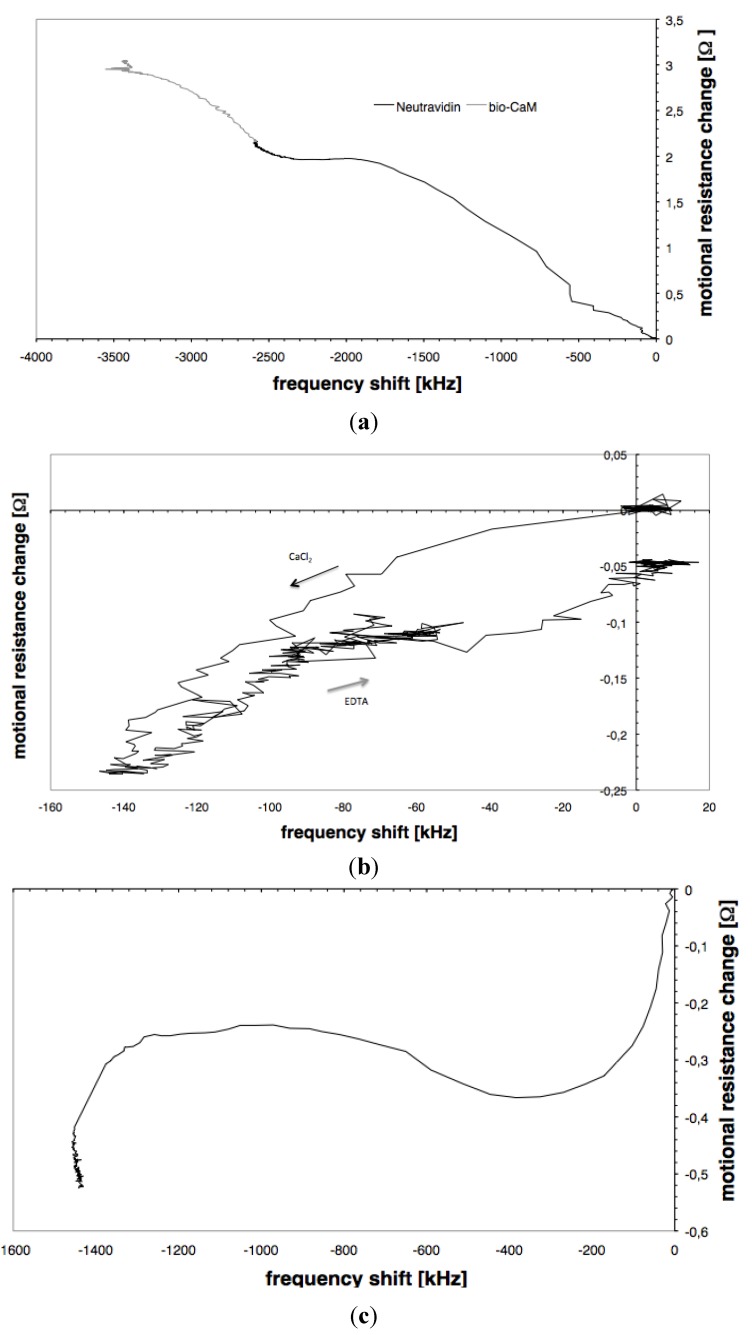
The adsorption of (**a**) Neutravidin and the binding of calmodulin, (**b**) the binding and de-binding of calcium to the calmodulin (**c**) and the peptide binding to the Ca^2+^/calmodulin plotted in resonant frequency *versus* motional resistance plots.

Figure 6(a) shows the adsorption of Neutravidin and the binding of biotinylated calmodulin to Neutravidin. Both adsorption processes are the binding of a soft material to the sensor surface. The mass of the soft biomolecules bound to the surface causes a frequency decrease and due to the viscoelastic character of the molecules, they dissipate acoustic energy thus increasing the motional resistance. In the motional resistance *versus* frequency plots the adsorption starts from the origin and proceeds into the second quadrant.

Binding of calcium to the calmodulin (for the highest calmodulin concentration) leads to a decrease in both frequency and resistance. The frequency decrease in this case is caused by the additional water coupled to the resonator, and the decrease in motional resistance is caused by a rigidification of the calmodulin. In the motional resistance *versus* frequency plots (Figure 6(b)) the adsorption starts from the origin and proceeds into the third quadrant. When calcium is bound to low surface densities of the calmodulin the curve extends into the fourth quadrant.

Figure 6(c) shows the plot for the peptide binding. Adsorption starts with a decrease in frequency and resistance, as observed with calcium adsorption indicating a conformational change. Then the curve turns towards an increase in motional resistance as found with the adsorption of Neutravidin (Figure 6(a)). At the end of the adsorption, the curve again indicates decreasing motional resistance. From the form of the curve we suggest that the mass adsorption is both preceded and followed by a conformational change of the calmodulin. 

While in the experiments by Furusawa *et al*. [15] two different types of processes—a conformational change and a mass adsorption—were identified, they were not able to see the initial conformational change clearly visible in our measurements. It might be that the reason for this difference is that the resonant frequency of the FBAR is at least one order of magnitude higher that the QCM used. The influence of attached mass, elasticity and viscosity of the adsorbent is highly dependent on the resonant frequency [36,37,38]. This means that certain changes of the adsorbent might be visible at one resonant frequency and not at another (higher or lower) resonant frequency. For a further investigation it might be interesting to simultaneously conduct the experiments of this paper with FBARs working at different resonant frequencies. 

## 4. Conclusions

In this paper we used the FBAR to monitor the conformational changes of calmodulin caused by calcium and peptide binding. Even though the limit of detection of this technology is higher than comparable technologies (e.g., SPR) and would be too high to detect the mass of the bound calcium, the signals caused by the conformational change were sufficient to determine an apparent *K_d_* for the binding. 

The dependence of the signals on the surface concentration shows the importance of a careful assay development in order to obtain accurate *K_d_* values. 

In addition to calcium adsorption, conformational changes were also discovered during the binding of a peptide to the Ca^2+^/calmodulin complex. During these assays, three different processes could be observed; the peptide binding (*i.e.*, a mass adsorption) and two conformational changes—one before, the other after the mass adsorption—could be distinguished. The different processes are more easily distinguished when plotting the motional resistance *versus* the resonant frequency shift and omitting the time axis. 

Judging from these first measurements investigating conformational changes, the FBAR is a promising transducer for the investigation of conformational changes in a high throughput format with low sample volume. However a better understanding of the FBAR behaviour is necessary in order to utilize this technology in routine measurements. In particular it needs to be clarified how coupled water, stiffening or other phenomena influence the FBAR measurement results. 
